# Maternal positive coparenting and adolescent ego-identity: the chain mediating role of fathers’ marital satisfaction and adolescent peer relationships

**DOI:** 10.3389/fpsyg.2023.1227941

**Published:** 2023-09-22

**Authors:** Wanghua Ji, Rui Ming Lan, Peng Ma, Hongpo Zhang, Lijun Fan

**Affiliations:** ^1^School of Management, Henan University of Chinese Medicine, Zhengzhou, China; ^2^School of Media and Exhibition, Fujian Business University, Fuzhou, Fujian, China; ^3^School of Psychology, South China Normal University, Guangzhou, Guangdong, China; ^4^College of Education, Huaibei Normal University, Huaibei, Anhui, China

**Keywords:** coparenting, ego-identity, marital satisfaction, peer relationships, adolescents

## Abstract

**Introduction:**

Based on the ecological systems theory and the family systems theory, this study explores the mechanisms underlying the effects of maternal positive coparenting on adolescent ego-identity.

**Methods:**

This study employed the Maternal Positive Coparenting Scale to assess mothers, the Father Marital Satisfaction Scale to examine fathers, and the Adolescent Peer Relationship Scale, along with the Ego-Identity Scale, to evaluate adolescents. This comprehensive approach involved investigating 522 families, encompassing both parents and adolescents.

**Results:**

The results obtained indicate a significant positive correlation between maternal positive coparenting and adolescent ego-identity. Peer relationships mediated the relationship between maternal positive coparenting and adolescent ego-identity. Father marital satisfaction mediated the relationship between maternal positive coparenting and adolescent ego-identity insignificantly. Paternal marital satisfaction and adolescent peer relationship have a chain mediating role between maternal positive coparenting and adolescent ego-identity. The study contributes by offering insights from the perspectives of family and peer relationships for further enhancing the development of adolescent ego-identity.

## 1. Introduction

Ego-identity refers to the individual’s thoughts and ideas about “who I am and how I define myself,” and is the subjective feeling and experience of an individual’s internal consistency and continuity (Fromm, 1968). Ego-identity is considered the most important psychological developmental task in the adolescent stage (Fromm, 1968; Chen et al., 2021; Maree, 2022). Good ego-identity development has been found to be associated with improved academic performance, social adjustment, and well-being among adolescents (Abu-Rayya, 2010; Pellerone et al., 2015; Perumal, 2020), whereas poor ego-identity development is linked to lower life satisfaction, hindered psychological health development, and even leads to problematic behaviors (Abu-Rayya, 2010; Waterman and Waterman, 2015; Saniye and Ayhan, 2023).

The family parenting environment has been recognized as the primary influence on adolescent ego-identity development (Bortz et al., 2019; Hasanah et al., 2019). Previous research has predominantly examined the impact of either the father’s (or the mother’s) parenting style or parent–child attachment on adolescents’ ego-identity (Grove, 2015; Zhang and Deng, 2015; Wang et al., 2017), overlooking the interactive dynamics among fathers, mothers, and children, such as the role of coparenting in adolescent ego-identity. Compared to one-sided father-child interactions between fathers and children or mothers and children, exploring more diverse father–mother–child interactions involving both fathers and mothers (e.g., coparenting) can provide a more comprehensive understanding of family dynamics and the influence of parents on children’s mental health development (Belsky and Hsieh, 1998; Schoppe-Sullivan et al., 2004). Furthermore, in the context of previous Western cultures, research on coparenting has primarily focused on divorced families (Beckmeyer et al., 2014; Bowers et al., 2014). In contrast to Western cultural norms, in Eastern cultures, nuclear families represent the predominant family structure. Within nuclear families, there exists the presence of spousal coparenting behaviors, where wives exhibit coparenting behaviors toward husbands, and husbands exhibit coparenting behaviors toward wives (Huang et al., 2019; Ji et al., 2022). With fathers increasingly engaging in coparenting, a mother’s positive attitude toward paternal coparenting assumes significant importance for family harmony and the psychological well-being of adolescents. However, there is currently a relative scarcity of research examining the influence of maternal positive coparenting on adolescents’ ego-identity development. Consequently, this study aims to delve deeply into the relationship between maternal positive coparenting and adolescent ego-identity and its underlying mechanisms. The findings of this study hold significant implications for enhancing our understanding of coparenting, refining coparenting theory, guiding family education practices, and promoting positive psychological development in adolescents.

### 1.1. Maternal positive coparenting and adolescents’ ego-identity

Coparenting refers to an alliance formed by parents during the process of raising children, encompassing positive or negative attitudes of one parent toward the other (Feinberg et al., 2007; Liu and Wu, 2015). Positive coparenting occurs when a family member responds positively to the child-rearing actions and goals of another family member. Based on this definition, maternal positive coparenting signifies the unity and consensus demonstrated by a mother in her child-rearing process toward the father’s parenting style (Ji et al., 2022).

The role of coparenting in adolescents’ psychological well-being has been widely acknowledged (Camisasca et al., 2019; Zhao et al., 2022), with different forms of coparenting exerting distinct effects on their psychological development. Supportive and positive coparenting between parents can enhance the parent–child relationship between parents and children, improve children’s adaptive emotional regulation abilities, increase positive emotions, and contribute to their psychological well-being (Thomassin et al., 2017; Coates et al., 2019). Conversely, destructive and negative forms of coparenting behaviors can lead to increased negative emotions, weakened emotional regulation abilities, and a greater susceptibility to mental health problems (Thomassin et al., 2017; Coates et al., 2019). Furthermore, research has demonstrated that supportive or destructive coparenting behavior from one parent toward the other can promote family intimacy and cohesion. Positive coparenting behavior by one parent toward the other can also increase adolescents’ sense of security and interpersonal trust (Chen and An, 2019; Huang et al., 2019).

While there is currently no direct evidence supporting a relationship between maternal positive coparenting and adolescent ego-identity, we can infer from the aforementioned studies that when a mother demonstrates a positive attitude and approach toward supporting the father’s parenting behavior (positive coparenting), it has the potential to enhance family intimacy and cohesion. This, in turn, may foster warmth and love experienced by adolescents, ultimately strengthening their sense of security and interpersonal trust. These factors are crucial variables that contribute to adolescent ego-identity development (Meeus et al., 2002; Wang et al., 2008; Årseth et al., 2009). Therefore, we propose hypothesis 1 (H1): Maternal positive coparenting is positively related to the level of adolescent ego-identity development.

### 1.2. The mediating role of fathers’ marital satisfaction

As proposed by Minuchin (1985), family systems theory posits that families are composed of a set of interacting subsystems, each interconnected subsystems that influence one another. The “crossover hypothesis,” informed by this theory, suggests that the emotions or behaviors of one family member within a family subsystem can impact the emotions or behaviors of another member in a different subsystem (Bolger et al., 1989; White, 1999). In the context of coparenting, this implies that mothers’ supportive and solidarity-based coparenting behaviors toward fathers can intersect with fathers’ attitudes toward marriage, such as their level of satisfaction.

Previous research has demonstrated that parental cooperation in child-rearing fosters closer relationships between parents, leading to increased marital satisfaction (Feinberg, 2003; Patrick et al., 2007; Morrill et al., 2010). Additionally, studies have examined the impact of coparenting on the psychological well-being of the other parent and have found that positive coparenting behavior from one parent toward the other can enhance positive psychological qualities, such as resilience and parenting efficacy, in the recipient parent (Tao, 2021). Building on these theoretical and empirical foundations, it can be inferred that positive coparenting behaviors exhibited by mothers in support and solidarity with fathers may improve parental intimacy and foster positive psychological qualities in fathers, thereby enhancing fathers’ satisfaction with marriage.

Similarly, according to the family systems theory spillover hypothesis of family systems theory, the parental relationship subsystem can also spill over and affect the child subsystem (Bolger et al., 1989; White, 1999). Fathers’ experiences with marriage can transfer to influence their children’s psychological development, including their ego identity. Research has shown that parents who are less satisfied with their own marriages are more likely to transfer this dissatisfaction to their children and adopt negative parenting styles (Coln et al., 2013), which negatively impact adolescents’ ego identity development (Wang et al., 2008). Furthermore, scholars have pointed out that parents in poor relationships experience and express more negative emotions in their daily lives, which can disrupt the parent–child relationship and lead to excessive control or a lack of family intimacy (Lindsey et al., 2009; Wang et al., 2016), hindering positive self-exploration and identity formation in adolescents. Therefore, this study formulates hypothesis 2 (H2): Father’s marital satisfaction plays a mediating role between maternal positive coparenting and adolescent ego-identity.

### 1.3. The mediating role of adolescent peer relationship

According to the ecological systems theory, the microsystem refers to the immediate environments of individual activities and interactions. During adolescence, both family and peer relationships constitute direct microenvironments that significantly influence the daily life and experiences of adolescents, holding vital implications for individual psychological development. Additionally, the mesosystem highlights the interconnectedness or relationships between microsystem environments. Consequently, an adolescent’s family environment, such as the parenting context, may be closely linked to their peer relationships (Harris, 1995; Brown et al., 1997). Social learning theory suggests that adolescents perceive their parents as role models and tend to imitate their behaviors and attitudes (Bandura, 1973). Positive coparenting behavior between parents can also be observed and learned by adolescents, and they may assimilate these behaviors into their own interpersonal communication skills, thus facilitating the establishment of positive peer relationships. Research has consistently shown that positive coparenting is associated with more positive social behavior and higher-quality peer attachments compared to negative coparenting (Leary and Katz, 2004; Huang et al., 2019). Therefore, it is expected that positive coparenting between mothers and fathers will have a positive impact on adolescent peer relationships.

Moreover, adolescent peer relationships, as a significant microsystem for adolescent psychological development, play a crucial role in adolescent ego-identity development. Becht et al. (2017) pointed out that there is a close association between peer relationships and the clarity of adolescents’ self-concept. The better the peer relationships, the clearer adolescents’ self-perception becomes. At the same time, peer acceptance provides emotional support and establishes a social network on which teenagers can depend during their journey of identity exploration (Zhang and Qin, 2023). Moreover, adolescents with strong peer relationships often receive more peer support and acceptance. Therefore, we propose hypothesis 3 (H3): Adolescent peer relationships mediate the relationship between maternal positive coparenting and adolescent ego-identity.

### 1.4. The chain-mediated role of paternal marital satisfaction and peer relationship

Based on the above analysis, it is proposed that a father’s marital satisfaction and peer relationships may serve as mediators between maternal positive coparenting and adolescent ego-identity. Similarly, based on the ecological systems theory, the family and peer relationships serve as two crucial microsystems in adolescent development. These two systems are not mutually exclusive; rather, they are interconnected and mutually influential (Pérez et al., 2021; Rivers et al., 2022). Therefore, father’s marital satisfaction, as a crucial variable in the family system, may also impact adolescent relationships with peers. Social learning theory suggests that children tend to develop their interpersonal communication patterns by observing their parents’ interaction behaviors (Bandura, 1973). When parents have low marital satisfaction, it often leads to more conflicts, arguments, and attacks (Rosen Grandon et al., 2004). Adolescents observe and learn these negative behaviors, assimilating them into their own interpersonal communication patterns, which hinders the establishment of positive peer relationships. On the other hand, when parents have high marital satisfaction, their interaction patterns tend to be more harmonious and intimate, which can help improve the quality of adolescent peer relationships.

**Figure 1:**
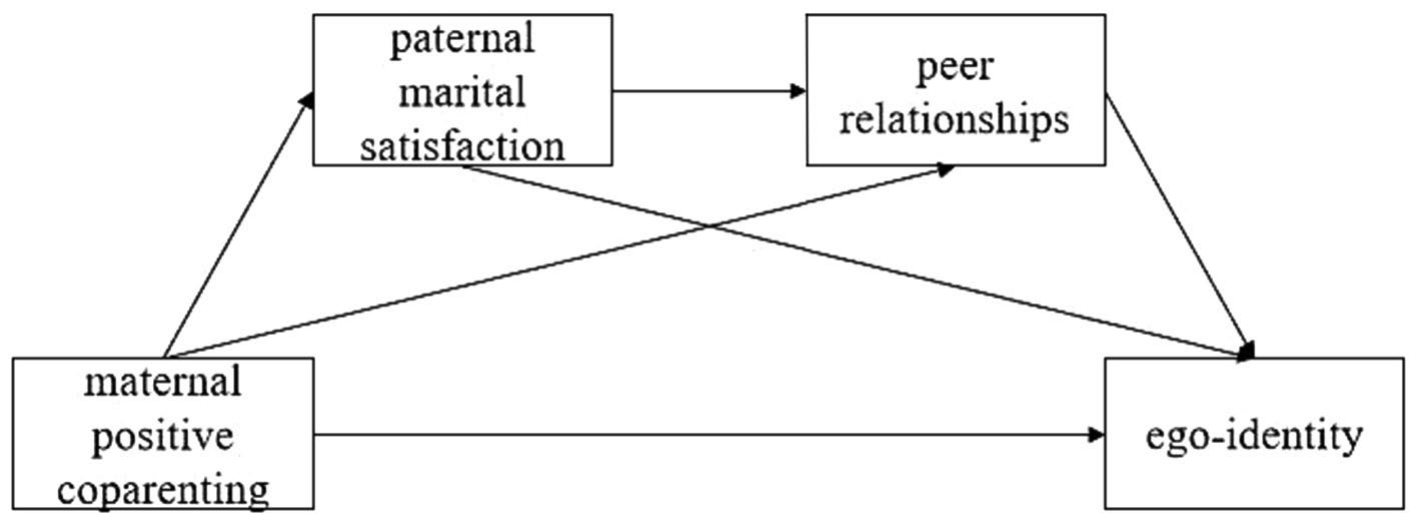
Hypothetical model diagram.

Empirical studies have shown that children growing up in families where parents are dissatisfied with their marital quality often exhibit higher levels of aggression or negative interpersonal communication (Buehler et al., 2009; Massar and Patil, 2020; Avci et al., 2021), hindering the establishment of positive relationships between adolescents and peers. Therefore, it is proposed that father’s marital satisfaction may positively predict the quality of adolescent relationships with peers. Finally, we propose hypothesis 4 (H4): Paternal marital satisfaction and peer relationships play a chain mediating role between maternal positive coparenting and adolescent ego-identity (Figure 1).

## 2. Methods

### 2.1. Setting and participants

The study utilized a whole-class sampling method and targeted middle school students in a specific area of Henan Province, China, as participants. The students and their parents were notified by their schools and invited to complete the questionnaires online through the platform Questionnaire Star platform. A total of 620 questionnaires were collected for the study. After excluding incomplete or duplicate responses, 522 sets of valid questionnaires were retained, which were completed by all three parties (i.e., fathers, mothers, and teenagers), resulting in a valid response rate of 84.19%. To eliminate the interference of parental divorce, data from 34 groups of divorced families were deleted subsequently, so 488 families were ultimately included in the study. Among the teenagers, there were 257 males and 231 females. Furthermore, the participants included 224 students in Grade 7, 165 students in Grade 8, and 99 students in Grade 9. The seventh-grade target adolescents ranged in age from 12 to 14 years (mean age 12.61), and 52.33% of them were girls. The fathers ranged in age from 31 to 65 years (mean age 41.58, *SD* = 5.08). The mothers ranged in age from 31 to 58 years (mean age 40.03, *SD* = 5.08).

### 2.2. Measures

#### 2.2.1. Maternal Positive Coparenting Scale

The Maternal Positive Coparenting Scale, originally developed by McHale (1997) and revised by Liu et al. (2017), was employed in this study. The scale comprises 17 items and is divided into two dimensions: Unity and Consistency. Participants rate each item on a 7-point scale, ranging from 1 (never) to 7 (always). Items were averaged and higher scores indicated a greater level of positive coparenting exhibited by the mother. Confirmatory factor analysis was conducted to assess the scale’s validity and yielded satisfactory results:*χ*^2^*/df* = 3.464, CFI = 0.928, TLI = 0.915, SRMR = 0.063, RMSEA = 0.069 [0.061, 0.076]. The α coefficient was 0.941.

#### 2.2.2. Father’s Marital Satisfaction Scale

The Father’s Marital Satisfaction Scale, initially developed by Fowers and Olson (1993), was utilized in this study. The scale is unidimensional and comprises 10 items. Participants rate each item on a 5-point scale. Responses are provided on this scale ranging from 1 (very inconsistent) to 5 (very consistent). Items were averaged, and higher scores indicating greater marital satisfaction in fathers. The fifth item was excluded from the scale due to a standardized factor loading of only 0.122. Confirmatory factor analysis yielded satisfactory results: χ^2^/df = 1.986, CFI = 0.983, TLI = 0.970, SRMR = 0.028, RMSEA = 0.043[0.024, 0.063]. The α coefficient was 0.841.

#### 2.2.3. Adolescent Peer Relationship Scale

The Peer Relationship Scale, developed by Damme et al. (2002), is a unidimensional scale comprising 10 items. The scale is scored on a 4-point scale, ranging from 1 (never) to 4 (always). Items were averaged and a higher score indicated better peer relationships for the child. Confirmatory factor analysis yielded satisfactory results: χ^2^/df = 3.772, CFI = 0.938, TLI = 0.912, SRMR = 0.083, RMSEA = 0.073 [0.059, 0.087]. The α coefficient was 0.846.

#### 2.2.4. Ego-Identity Scale

The Ego-Identity Scale, revised by Zhang (2000), was employed in this study. The scale comprises 12 items and is scored on a 6-point scale, ranging from 1 (very inconsistent) to 6 (very consistent). Items were averaged and a higher score indicated a higher level of ego-identity. Confirmatory factor analysis yielded satisfactory results: χ^2^/df = 2.424, CFI = 0.971, TLI = 0.944, SRMR = 0.037, RMSEA = 0.052[0.035, 0.070]. The α coefficient was 0.789.

### 2.3. Data analysis

Descriptive statistics, reliability analysis, and common method bias analysis were conducted using SPSS 25. Confirmatory factor analysis, path analysis, and structural equation modeling were performed using Mplus 8.3.

## 3. Results

### 3.1. Common method bias analysis

Harman’s single-factor test was conducted by performing an unrotated factor analysis on the items of the respective scales. The results indicate that nine factors had eigenvalues greater than 1. The first factor accounted for 20.745% of the variance, below the critical threshold of 40%. Therefore, it can be concluded that the presence of common method bias is insignificant.

### 3.2. Descriptive statistics results

Correlation analysis was conducted among the total scores of the variables. The results indicated that the associations between family socioeconomic status, parental education level, and other variables are relatively low, and according, they will not have a significant impact on the model. Therefore, these variables will not be included in the subsequent covariate analysis. The results of correlation analysis showed that the correlation coefficients between the total scores of maternal positive coparenting, father’s marital satisfaction, adolescent peer relationship and ego-identity ranged from 0.127 to 0.436 (all *p* < 0.01). Due to the complexity of the measurement model, a parceling technique was adopted to construct the structural equation model, which retained the information of the original items and dimensions while achieving acceptable model fit. Mother’s positive coparenting and ego-identity were parceled based on their respective dimensions to enhance common variance and reduce random error. The factor loading of the 5th item in paternal marital satisfaction was only 0.122, leading to its removal. The remaining items were parceled into 3 groups using a high-loading approach to increase indicator consistency. As for adolescent peer relationships, since the number of reverse-scored items was equal to the number of forward-scored items, a unique information approach was used to parcel them into 2 groups, which helped to reduce within-group differences (Table 1).

**Table 1:** Descriptive statistics and correlation analysis of total scores for each variable (*n* = 488).

Index	M ± SD	1	2	3	4	4	5	6
1. Family socioeconomic status	4.865 ± 2.096	1						
2. Maternal education level	2.750 ± 1.176	0.280**	1					
3. Maternal education level	2.994 ± 1.202	0.329**	0.614**	1				
4. Maternal positive coparenting	72.207 ± 19.893	0.072	0.111*	0.107*	1			
5. Paternal marital satisfaction	39.033 ± 6.643	0.105*	0.055	0.084	0.170**	1		
6. Peer relationships	31.996 ± 5.506	−0.066	−0.001	0.007	0.253**	0.143**	1	
7. Ego-identity	47.992 ± 8.170	0.075	0.080	0.131**	0.131**	0.307**	0.137**	0.424**

*p < 0.05, **p < 0.01, ***p < 0.001.

### 3.3. The structural equation model testing

**Figure 2:**
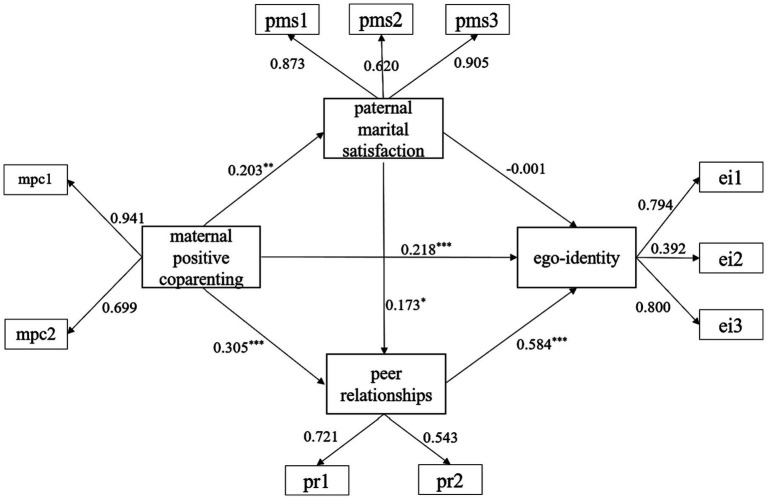
Chain mediated regression path. *p < 0.05, **p < 0.01, ***p < 0.001.

The structural equation model was constructed using the parceling groups, and the results indicated a good model fit: *χ*^2^*/df* = 1.372, CFI = 0.995, TLI = 0.992, SRMR = 0.025, RMSEA = 0.027 [0.000, 0.046]. The path coefficients of the mediated pathways were examined, and the results are presented in Table 2. The predictive effect of maternal positive coparenting on paternal marital satisfaction was significant (coeff = 0.185, *z* = 3.442, *p* < 0.001). The predictive effect of maternal positive coparenting on peer relationships was significant (coeff = 0.265, *z* = 3.598, *p* < 0.001), as well as the predictive effect of paternal marital satisfaction on peer relationships (coeff = 0.133, *z* = 2.413, *p* < 0.05). The predictive effect of maternal positive coparenting on ego-identity was significant (coeff = 0.210, *z* = 3.720, *p* < 0.001), while the predictive effect of paternal marital satisfaction on ego-identity was not significant (coeff = 0.018, *z* = 0.379, *p* > 0.05). The predictive effect of peer relationships on ego-identity was significant (coeff = 0.615, *z* = 9.555, *p* < 0.001) (Figure 2).

**Table 2:** Analysis of chain mediation path coefficients.

Model	Outcome variable	Predictive variable	Coefficient	Est./S.E.
1	Paternal marital satisfaction	Maternal positive coparenting	0.203	3.790***
2	Peer relationships	Maternal positive coparenting	0.305	3.920***
		Paternal marital satisfaction	0.173	2.703**
3	Ego-identity	Maternal positive coparenting	0.218	3.007**
		Paternal marital satisfaction	−0.001	−0.014
		Peer relationships	0.584	7.197***

*p < 0.05, **p < 0.01, ***p < 0.001.

Further mediation analysis revealed that the mediating effect of paternal marital satisfaction was not significant (*coeff* = 0.003, *p* > 0.05), while all other effects were significant (all *p* < 0.05), and the confidence intervals did not include 0. Specifically, the direct effect (0.210), the mediating effect of peer relationships (0.163), and the chain-mediated effect of paternal marital satisfaction and peer relationships (0.015) accounted for 53.708, 41.688, and 3.836%, respectively, of the total effect (0.391). Specific details can be found in Table 3.

**Table 3:** Decomposition of mediation effects.

	Effect	Est./S.E.	Lower 2.5%	Upper 2.5%	Percentage
Direct effect	0.218	3.007***	0.069	0.3329	52.163%
Indirect effect	0.199	3.335**	0.105	0.318	47.837%
M1	0.000	−0.013	−0.024	0.017	0.000%
M2	0.178	3.026**	0.087	0.294	42.788%
M3	0.021	1.965*	0.006	0.044	5.048%
Total effect	0.416	8.119***	0.312	0.498	–

M1, Maternal positive coparenting→Paternal marital satisfaction→Ego-identity; M2, Maternal positive coparenting→Peer relationships→Ego-identity; M3, Maternal positive coparenting→Paternal marital satisfaction→Peer relationships→Ego-identity; Lower 2.5% and Upper 2.5% indicate BCBootstrap 2.5 and 97.5%. *p < 0.05, **p < 0.01, ***p < 0.001.

## 4. Discussion

### 4.1. Maternal positive coparenting and adolescents’ ego-identity

This study provides innovative insights into the relationship between maternal positive coparenting and adolescents’ ego-identity. The findings support the research hypothesis by demonstrating that maternal positive coparenting positively predicts the level of adolescents’ ego-identity. The results emphasize the significance of maternal positive coparenting as a significant family variable associated with the development of adolescents’ ego-identity. Mothers adopting a unified and positive coparenting approach with fathers fosters intimacy and cohesion within the family. Consequently, adolescents to feel more supported, and secure, and experience warmth within the family context (Chen and An, 2019; Huang et al., 2019). These factors contribute to the healthy development of adolescents’ ego-identity (Meeus et al., 2002; Wang et al., 2008; Årseth et al., 2009). Conversely, lower levels of positive coparenting may result in reduced intimacy among family members and increased family conflicts, which can hinder the positive development of adolescents’ self-awareness and ego-identity.

### 4.2. The mediating role of fathers’ marital satisfaction

The results indicate that parental marital satisfaction does not significantly mediate the relationship between maternal positive coparenting and adolescent ego-identity. However, the analysis revealed that maternal positive coparenting is positively associated with father’s marital satisfaction. This finding is consistent with prior research (Feinberg, 2003; Morrill et al., 2010), suggesting that when mothers demonstrate a consistent and united approach to fathers’ parenting, it promotes a higher level of intimacy between the parents. These findings further validate the Family Systems Theory, which suggests that multiple subsystems within a family system interact and rely on each other (Minuchin, 1985). The triadic coparenting subsystem, comprising the father, mother, and child, can also “spillover” and impact the behavior or attitudes of the parent–child subsystem (father’s marital satisfaction).

It is worthy to note that father’s marital satisfaction cannot directly predict adolescents’ levels of ego-identity. This finding further validates the coexistence of the spillover and compensation hypotheses within the Family Systems Theory. According to the Family Systems Theory, various subsystems within the family (such as the father-child, mother–child, and marital subsystem) mutually influence each other, following the spillover or compensation hypotheses (Erel and Burman, 1995). Based on the spillover hypothesis, lower marital satisfaction in fathers indicates more conflicts, dissatisfaction, and negative emotional and behavioral interaction patterns within the marital relationship (Wang et al., 2016). These negative patterns and emotions may potentially spill over into the father-child interaction process, resulting in more negative interactions with adolescents (Coln et al., 2013), which hinders adolescents’ self-exploration and commitment, thus impairing the development of adolescents’ self-identity (Meeus et al., 2002). On the other hand, according to the compensation hypothesis (Erel and Burman, 1995), when fathers have lower marital satisfaction and their need for intimate relationships within the marital relationship is not fulfilled, they may redirect the missing marital intimacy toward their adolescents by providing more attention and emotional support. This further promotes adolescents’ positive self-exploration and self-identity development (Wang et al., 2008). Therefore, due to the simultaneous spillover and compensation effects, the promotion effect of father’s marital satisfaction on adolescents’ self-identity is not significant in the results. However, further research can explore potential moderating variables, such as the father’s personality type (de Moor et al., 2019), to investigate why some fathers follow the spillover hypothesis while others follow the compensation hypothesis.

### 4.3. The mediating role of adolescent peer relationships

The research results indicate that adolescent peer relationships mediate the relationship between maternal positive coparenting and adolescent ego-identity, thus supporting Hypothesis 3. According to the ecological systems theory, family dynamics such as parental coparenting and peer relationships serve as crucial micro-level environments for adolescent psychological development, including aspects like ego-identity. Furthermore, the mesosystem emphasizes the interconnectedness and interrelations between various micro-level systems. As a result, the coparenting within the family environment and adolescent peer relationships are likely to be closely interconnected in the context of adolescent development (Zhang and Qin, 2023). When there is supportive and cohesive coparenting within the family, it enhances adolescents’ sense of security and interpersonal trust, leading to improved attachment quality with their peers and facilitating the formation of positive peer relationships (McHale, 1997; Chen and An, 2019; Huang et al., 2019). Simultaneously, engaging in peer interactions is beneficial for adolescents’ self-identity development (Kerpelman et al., 2012; Becht et al., 2017). The process of communication with adolescent peers provides opportunities for self-identification. Adolescents receive acceptance and support from positive peer relationships, which facilitates their active self-exploration (Newman and Newman, 1976; Weeks and Pasupathi, 2010). As a result, this promotes the development of adolescents’ ego-identity levels.

### 4.4. The chain mediating role of fathers’ marital satisfaction and adolescent peer relationships

This study also found that the mediating pathway of “father’s marital satisfaction → peer relationships” is an important mechanism through which maternal positive coparenting is associated with adolescent ego-identity, supporting Hypothesis 4. This result suggests that a mother’s supportive and consistent attitude or behavior toward coparenting with the father can enhance the intimacy among parents (Feinberg, 2003; Morrill et al., 2010), thereby increasing the father’s satisfaction with marriage (Patrick et al., 2007). Higher levels of paternal marital satisfaction may be associated with less family conflict and more positive patterns of husband-wife interactions which may be imitated and learned by adolescents (Wu et al., 2016; Becht et al., 2017), thus influencing the quality of adolescent peer relationships (Cui et al., 2018); Communication with peers can further promote adolescent identity formation and self-exploration (Weeks and Pasupathi, 2010; Becht et al., 2017), thus promoting the development of adolescent ego-identity. Therefore, the mediating pathway of “father’s marital satisfaction → peer relationships” serves as an important bridge between a maternal positive coparenting and adolescent ego-identity. This finding confirms the perspective of the ecological systems theory, where both family and peers are core microsystems in adolescent development, interconnected and able to jointly affect adolescent psychological development (Pérez et al., 2021; Rivers et al., 2022).

### 4.5. Research significance

This study investigates the effect of maternal positive coparenting, paternal marital satisfaction, and peer relationships on adolescent ego-identity from family system theory and ecological systems theory perspectives. It holds both theoretical and practical significance.

Firstly, the study’s sample was selected from intact nuclear families within an Eastern cultural context. The findings of this research offer empirical references for potential cross-cultural investigations into co-parenting dynamics, spanning both Eastern and Western environments. Moreover, these results enrich the content and significance of family systems theory.

Furthermore, the findings of this study also hold significant educational implications for enhancing adolescent ego-identity. Firstly, The findings of this study contribute to enhancing adolescent ego-identity from the perspectives of psychological counseling practices and the collaboration between home and school. In the process of psychological counseling, it is beneficial to guide adolescents’ mothers in increasing their positive coparenting behaviors. Simultaneously, within the school environment, encouraging teachers to facilitate the formation of positive peer relationships among adolescents can further elevate their ego-identity; In the cultural context of China, as primary caregivers, mothers should aim for a cohesive and consistent approach to involving fathers in parenting. Secondly, when mothers engage with their children, providing positive feedback to fathers’ parenting behaviors, it results in an increase in paternal marital satisfaction. This, in turn, encourages adolescents to learn positive interaction patterns from their parents, further enhancing their peer relationships. Consequently, this progression is conducive to the development of adolescent ego-identity.

### 4.6. Research limitations and prospects

While this study provides theoretical and empirical support for exploring the association between maternal positive coparenting and adolescent ego-identity, several limitations should be addressed in future research.

Firstly, this study has not taken into account potential moderating variables in the relationship pathway between maternal positive coparenting and adolescent ego-identity. However, family life factors, such as unemployment, economic preferences, and education level, which are crucial variables within the microsystem of the family, might serve as latent moderating variables within the pathway between maternal positive coparenting and adolescent ego-identity. Therefore, in future research, investigating these moderating variables and family life factors could provide greater depth to our insights.

Secondly, the results of the mediation analysis in this study revealed that the mediating variables played a partial mediating role in the relationship between maternal positive coparenting and adolescent ego-identity, rather than a full mediation. Therefore, future research could continue to explore the influence of other potential mediating variables, such as family life factors. This could provide a deeper understanding of the pathways that underlie the relationship between maternal positive coparenting and adolescent ego-identity.

Thirdly, this study employs a quantitative research approach for investigation. In the future, it is advisable to consider incorporating qualitative research methods, such as interviews, to explore the experiences of parents and adolescents during their growth that could potentially impact the development of adolescent ego-identity.

Fourthly, this study employed a cross-sectional design, which limits the ability to make causal inferences about the relationships between variables. Future longitudinal studies could examine the dynamic nature of the relationship between maternal positive coparenting and adolescent ego-identity over time.

Lastly, the sample for this study comprised only families from central cities in China. It thus remains unclear whether the findings can be generalized to economically more developed coastal cities. Future research could possibly expand the sample size and consider surveys conducted in economically developed coastal areas to further explore the generalizability of the results.

## 5. Conclusion

From the perspective of positive psychology, based on family system theory and family-peer linkage perspectives, this study investigated the mechanism of adolescent ego-identity formation. The findings suggest that there is an association between maternal positive coparenting and adolescent ego-identity, with maternal positive coparenting being linked not only to direct predictions of adolescent ego-identity but also to indirect predictions through adolescent peer relationships or the “paternal marital satisfaction → peer relationships” pathway.

## Data availability statement

The raw data supporting the conclusions of this article will be made available by the authors, without undue reservation.

## Ethics statement

The studies involving humans were approved by the Research Ethics Committee of the Institute of Psychology and Behavior, Henan University. The studies were conducted in accordance with the local legislation and institutional requirements. Written informed consent for participation in this study was provided by the participants’ legal guardians/next of kin.

## Author contributions

WJ: conceptualization, methodology, data curation and writing-review and editing. RL: writing-original draft, review and editing. PM: data curation, formal analysis. HZ: validation and investigation. LF: data collection, editing. All authors contributed to the article and approved the submitted version.
